# Investigating the prevalence and associated factors of depression, anxiety, and loneliness among people with type-2 diabetes in Bangladesh: a community-based study

**DOI:** 10.1038/s41598-024-75144-3

**Published:** 2024-10-24

**Authors:** Shahina Pardhan, Abu Bakkar Siddique, Umma Motahara, Md. Saiful Islam

**Affiliations:** 1 Vision and Eye Research Institute, School of Medicine, Anglia Ruskin University, Young Street, Cambridge, UK; 2 Centre for Inclusive Community Eye Health, School of Medicine, Anglia Ruskin University, Young Street, Cambridge, UK; 3 Department of Public Health and Informatics, Jahangirnagar University, Savar, Dhaka, 1342 Bangladesh; 4 Department of International Health, Johns Hopkins Bloomberg School of Public Health, Baltimore, MD 21205 USA

**Keywords:** Depression, Anxiety, Loneliness, Sociodemographic factors, Bangladesh, Human behaviour, Psychology, Risk factors

## Abstract

Diabetes mellitus is a major worldwide health concern. Diabetes has been associated with a number of adverse mental health conditions including depression, anxiety, and loneliness that can negatively impact diabetes outcomes. This study aimed to investigate factors associated with depression, anxiety, and loneliness in people, in the community, suffering with diabetes in Bangladesh. A cross-sectional study was conducted with 600 people with type-2 diabetes (54.83% females; mean age: 52.70 ± 11.56 years) between July and September 2022. Purposive sampling method was used to recruit the participants. A validated semi-structured questionnaire was used to collect demographic and other data. Depression, anxiety, and loneliness were measured using the PHQ-9, GAD-7, and UCLA Loneliness scale, respectively. Bivariate and multivariable linear regression analyses were conducted to ascertain factors that were significantly associated with these mental health conditions. The prevalence of depression, anxiety, and loneliness was 31.17%, 21.83%, and 28.00%, respectively. A lack of formal education, and not taking part in physical activities were significantly associated with all three mental health states. Duration of diabetes and being on medication for high cholesterol were also associated with depression and anxiety. Older age and being widowed were significantly associated with loneliness. This study found that depression, anxiety, and loneliness are prevalent among Bangladeshi people with diabetes, with certain sociodemographic and diabetes-related factors associated with increased risk. The findings emphasize the need for targeted interventions to people within the communities, at grassroot levels in order to improve reduce health inequality, and improve the mental health of people living with diabetes.

## Introduction

Diabetes mellitus (DM) is a metabolic disorder characterized by hyperglycemia caused by insulin secretion defects^1^. DM is prevalent across the world, with prevalence rates ranging by area, and between rural and urban populations^2^. The World Health Organization (WHO) estimates that around 422 million individuals worldwide have diabetes^3^. Thus, DM is recognized as a major worldwide health issue^2^. The International Diabetes Federation (IDF) predicts that 700 million will be affected by 2045 and according to the IDF, 79% of those with diabetes reside in low- and middle-income countries (LMICs)^4^. In Bangladesh, the prevalence of diabetes stands at 9.2%, with rates among men at 8.8% and among women at 9.6%. Additionally, prediabetes affects 13.3% of the population, with rates among men at 13.0% and among women at 13.6%^5^. The prevalence of diabetes is expected to rise by 74% in Southeast Asian nations over the next two decades, from 88 million in 2019 to 153 million by 2045^6^. In 2021, there were 13.1 million people living with diabetes and the country was ranked as the 8th highest international. It is predicted that this will increase to being the seventh highest with a prevalence of 43 million by 2030^5^. In terms of people of older age, Bangladesh was rated eighth with the largest number of adults (20–79 years) with diabetes by the IDF in 2021^5^. Depression is a widespread mental condition that has a detrimental influence on productivity and quality of life^7^. Evidence shows significant associations between depression and diabetes^8^. A meta-analysis estimated the prevalence of depression among diabetics to be twice that of the general population^9^. Anxiety and other mental health conditions have also been shown to be highly prevalent in people associated with diabetes^10^, with both anxiety and depression are linked to worse diabetes outcomes^10,11^. A systematic review and meta-analyses revealed a significant correlation between diabetes and anxiety symptoms and disorders^12^. Loneliness has also been associated with general morbidity and mortality in the adult population^13^, and has been linked to a number of chronic illnesses including heart disease, hypertension, stroke, obesity, diabetes, and pulmonary disease^14^. Diabetes and the higher risk of reduced mental health have been linked to various physical and psychological factors^15^. The chronic nature of diabetes requires daily management is linked to increased stress and emotional strain, and a diminished quality of life. Anxiety around possible long-term complications would contribute to decreased mental wellbeing, needing systemic and psychological care in people with diabetes^16,17^. Systemic inflammation is a significant risk factor for the poor health outcomes connected to diabetes, which in turn has been linked to increased stress^18^. It has also been shown that fluctuations in blood glucose levels can impact mood and cognitive function, contributing to anxiety and depression^15,19^. There is a paucity of research on depression, anxiety, and loneliness in Bangladesh^20^. While there are a number of prior studies^21,22^ that have examined mental wellbeing in patients with diabetes in hospital setting, and individual with chronic medical conditions (e.g., diabetes, hypertension, obesity, heart disease, etc.). To our knowledge, there are no studies that have compared depression, anxiety, and loneliness together in the same cohort of individuals in a community setting. This is quite important as the profile of people within the community may be quite different when compared to those who attend hospitals. It is envisaged that the results of the study would enable us to identify the factors that are significantly associated with depression, anxiety, and loneliness at grassroot levels, in order to inform policy makers and healthcare practitioners in planning, and developing strategies in order to improve the mental wellbeing of people living with diabetes in the community.

## Methodology

### Study design and setting

The current study employed a cross-sectional and interview-based survey of Bangladeshi people living with type-2 diabetes. Data were collected from the central region (Gopalganj and Jhenaidah districts) and the western region (Joypurhat and Dinajpur districts) of Bangladesh in the community between July and September 2022, via a door-to-door visit.

### Study procedure

Validated questionnaires on depression, anxiety, and loneliness were used to collect data from the face-to-face interviews conducted^23–25^. The questionnaires were translated from English to Bengali by a dual language expert which was then translated back to English (by another dual language expert to ensure validity, which was then compared and checked by the lead investigator and supervisor. A pilot test was carried out on 10 participants from the same population (target group) to determine the acceptability and transparency of the questionnaire. Following this, a few minor adjustments were made to the questionnaire, mostly around some of the terms used. The questionnaire was translated back into English and validated. Data obtained from the pilot testing were excluded from the final analysis.

Data were collected using purposive sampling. The data collectors asked the household members about their diabetes status. If they responded ‘yes’, then the interviews were conducted. The questionnaire included informed consent statement that explained the study’s objectives, procedures, and the participant’s right to decline if they so wished. The inclusion criteria of the participants included: i) being adults (≥ 18 years), ii) suffering from DM for at least 6 months, iii) being Bangladeshi residents, and iv) having willingness to take part in the survey.

### Sample size

The sample size was calculated using the following equation:

$$\:n=\frac{{z}^{2}pq}{{d}^{2}};\:n=\frac{{1.96}^{2}\times\:0.5\times\:\left(1-0.5\right)}{{0.05}^{2}}=384.16\approx\:384$$

Here,

*n* = number of samples.

*z* = 1.96 (95% confidence level).

*p* = prevalence estimate (50% or 0.5) (as no study found).

*q* = (1-*p*).

*d* = Precession of the prevalence estimate (10% of 0.05).

Considering a 10% non-response rate, a sample size of 423.5 (rounded up to 424 people), was estimated. Our sample size exceeded this value as 607 individuals were interviewed, after obtaining informed consent, using a purposive sample method. Missing or incomplete data of seven participants were discarded, leaving 600 in the final analysis.

### Measures

#### Socio-demographic measures

Socio-demographic data included age, sex (male/ female), marital status (married/ unmarried/ separated or widowed), education (no formal education, primary, secondary, university), monthly family income (< 15,000 Bangladeshi Taka [BDT] [< 136.69 US$]/ 15,000–30,000 BDT [136.69-273.39 US$]/ >30,000 BDT [> 273.39 US$]) (GDP per capita 2,688.3 in Bangladesh [in 2022]^26^), employment status (employed/ unemployed/ self-employed), and family type (nuclear/ joint).

#### Diabetes and lifestyle measures

Data were obtained on duration of diabetes (in years), medication for high blood pressure (yes/ no), medication for high cholesterol (yes/ no), cigarette smoking (yes/ no), and whether participants took part in physical exercise (yes/ no).

### Patient health questionnaire for depression (PHQ-9)

The PHQ-9 scale assesses the level of depression and also the onset of depressive illness^27^. The translated Bangla Patient Health Questionnaire’s 9-item scale was used^23^ and responses to the 9 items were recorded on a four-point Likert scale that ranged from 0 (“Never”) to 3 (“Almost every day”). The scores were categorized into five groups according to the severity: minimum (0–4), mild (5–9), moderate (10–14), moderately severe (15–19), and severe (20–27)^28^. Individuals scoring within the moderate to severe range (scores ≥ 10) were classified as suffering from depression^28^, in line with previous studies^29–31^. The Cronbach’s alpha of the PHQ-19 was 0.89, indicating very good reliability.

### Generalized anxiety disorder (GAD-7)

The GAD-7 scale was used to screen anxiety and grade its severity^32^. The GAD-7 scale^24^ consists of seven items that are answered on a four-point Likert scale ranging from 0 (“Never”) to 3 (“Almost every day”). This was translated into Bengali as explained above. Based on scores the level of anxiety was divided into four categories: low (0–4), mild (5–9), moderate (10–14) and severe (15–21). Individuals with scores equal to and greater than 10 (moderate to severe) were classified as suffering from anxiety^32,33^, in line with previous studies^34,35^. The Cronbach’s alpha of the GAD-7 was 0.88, indicating very good reliability.

### University of California, Los Angeles (UCLA) loneliness scale

The Bengali-translated UCLA scale was used to measure participants’ loneliness^25,36^. Three items with three-point Likert scales ranged from 1 (“Hardly Ever”) to 3 (“Often”) for three questions *“How often do you feel that you lack companionship?”*,* “How often do you feel left out?”*,* and “How often do you feel isolated from others?”*). The total score was calculated by adding scores ranging from 3 to 9, with higher values indicating greater loneliness. A cutoff (≥ 6) was used to determine significant loneliness in line with previous work^36–38^. The Cronbach’s alpha of the UCLA was 0.84 in the current study, indicating good reliability.

### Statistical analysis

Data were analyzed using Statistical Package for Microsoft Excel (version 2019), Social Sciences (SPSS version 25.0), and STATA (version 15.0). Descriptive statistics (i.e., frequencies, percentages, means, standard deviations) were computed. To examine any association between dependent and independent variables, bivariate linear regression analysis and multivariable linear regression analysis (on significant associations from bivariate analysis) were performed. A *p*-value less than 0.05 was deemed as statistically significant for all analyses.

### Ethical approval

The study protocol was reviewed and approved by the Ethical Review Committee, Uttara Adhunik Medical College, Uttara, Dhaka-1230, Bangladesh [Ref. No.: UAMC/ERC/03/2022/12]. All procedures of the present study were conducted in accordance with human involving research guidelines (e.g., Helsinki Declaration). Informed consent was obtained from each participant after the study’s procedures, objectives, and confidentiality about their information, etc., were discussed and clearly documented. The data were collected anonymously and analyzed using numerical codes.

## Results

### General characteristics of the participants

A total of 600 participants with a mean age of 52.70 years (SD = 11.56) were included in the final analysis. Of these, 55% were female and a large proportion were married (79%) (Table 1). Less than half of the individuals had attained at least primary level of education (45%), 46% were self-employed, and 41% reported that their monthly family income was more than 30,000 BDT.

**Table 1:** Distribution of the study variables (*N* = 600).

Variables	*n* (%)
Age (mean ± SD)	52.70 ± 11.56
Gender	
Male	271 (45.17)
Female	329 (54.83)
Education	
No formal education	147 (24.5)
Primary	270 (45)
Secondary	1 (0.17)
University	182 (30.33)
Marital status	
Unmarried	35 (5.83)
Married	471 (78.5)
Separated	10 (1.67)
Widowed	84 (14)
Employment status	
Employed	158 (26.33)
Unemployed	167 (27.83)
Self-employed	275 (45.83)
Monthly family income	
< 15,000 BDT	191 (31.83)
15,000–30,000 BDT	162 (27)
> 30,000 BDT	247 (41.17)
Duration of diabetes (mean ± SD)	6.60 ± 6.21
Taking medication for high blood pressure	
Yes	405 (67.5)
No	195 (32.5)
Taking medication for high cholesterol	
Yes	300 (50)
No	300 (50)
Cigarette smoking	
Yes	180 (30)
No	420 (70)
Physical exercise	
Yes	521 (86.83)
No	79 (13.17)

The participants’ mean duration of diabetes was 6.60 years (SD = 6.21). A high proportion (68%) reported to taking medication for high blood pressure, and 50% for high cholesterol. 30% reported to smoking cigarettes and 86% reported to take part in some physical activity.

### Prevalence and associated factors of depression

**Fig. 1:**
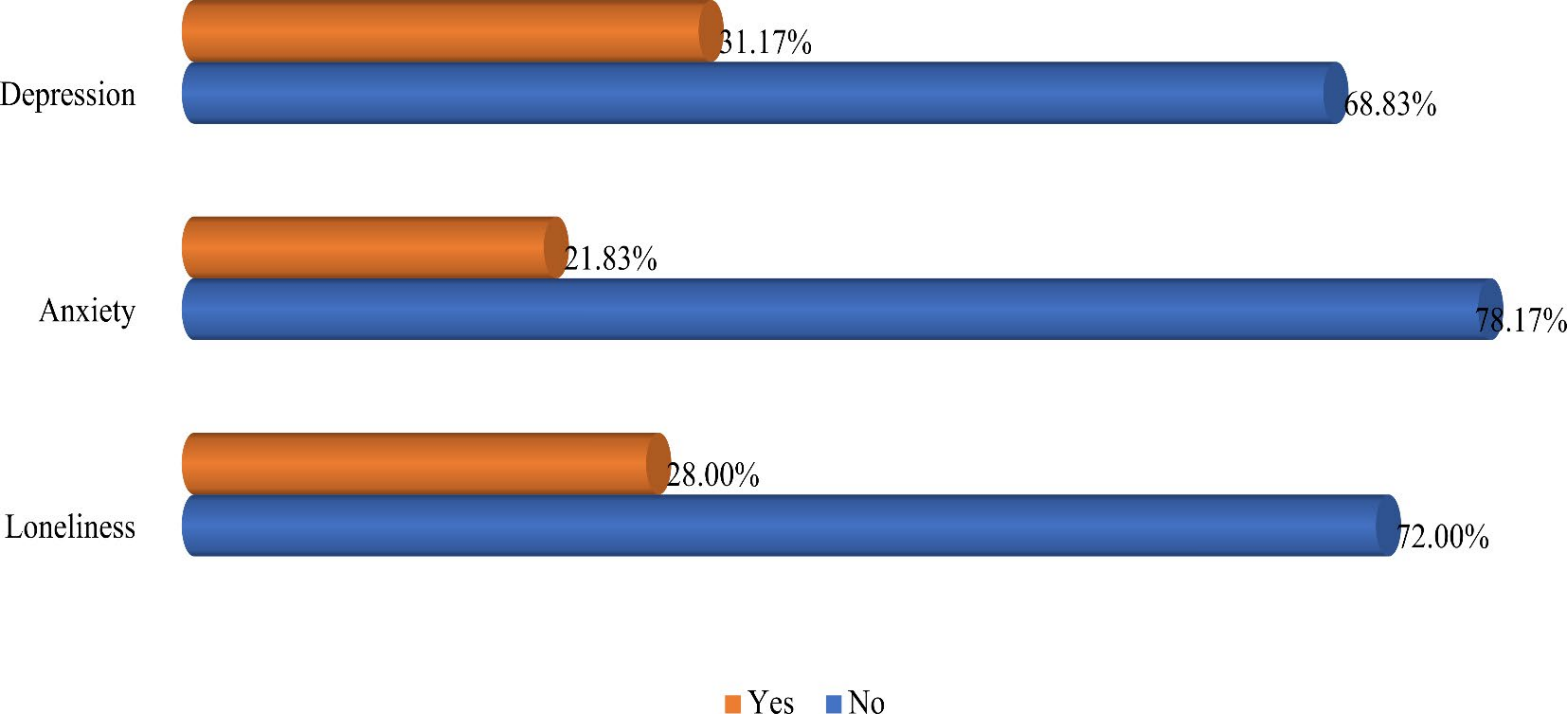
Prevalence estimates of depression, anxiety, and loneliness.

The prevalence of moderate to severe depression was 31% among the participants (Fig. 1). Multivariable regression analysis showed significant association of depression with no formal education (β = 0.16, *p* = 0.007), being unemployed (β = 0.13, *p* = 0.013), duration of diabetes (β = 0.11, *p* = 0.012), taking medication for high cholesterol (β = 0.11, *p* = 0.015), and not taking part in any physical activity (β = 0.18, *p* < 0.001) (Table 2).

**Table 2:** Bivariate and multivariable linear regression analysis predicting depression.

Variables	Mean (SD)	Bivariate regression analysis					Multivariable regression analysis				
		B	SE	t	β	*p*-Value	B	SE	t	β	*p*-Value
Age	−	0.08	0.02	4.53	0.18	**< 0.001**	0.04	0.02	1.65	0.08	0.099
Gender											
Female	8.45 (5.22)	1.19	0.43	2.77	0.11	**0.006**	0.71	0.43	1.63	0.07	0.103
Male	7.25 (5.27)				Ref.					Ref.	
Education											
No formal education	9.76 (5.74)	2.70	0.57	4.71	0.22	**< 0.001**	1.92	0.71	2.70	0.16	**0.007**
Primary	7.49 (4.88)	0.43	0.50	0.87	0.04	0.382	0.52	0.57	0.93	0.05	0.354
Secondary	5.00 (0.00)	-2.06	5.19	-0.40	-0.02	0.692	-2.07	4.93	-0.42	-0.02	0.675
University	7.05 (5.12)				Ref.					Ref.	
Marital status											
Unmarried	7.94 (4.84)	0.34	0.92	0.37	0.02	0.708	0.34	0.88	0.39	0.02	0.697
Separated	10.5 (7.34)	2.90	1.68	1.73	0.07	0.084	1.18	1.58	0.75	0.03	0.457
Widowed	9.32 (5.51)	1.72	0.62	2.78	0.11	**0.006**	-0.09	0.64	-0.13	-0.01	0.893
Married	7.60 (5.17)				Ref.					Ref.	
Employment status											
Unemployed	9.32 (5.18)	2.75	0.58	4.79	0.23	**< 0.001**	1.52	0.61	2.50	0.13	**0.013**
Self-employed	7.83 (5.19)	1.26	0.52	2.44	0.12	**0.015**	0.35	0.60	0.59	0.03	0.557
Employed	6.56 (5.17)				Ref.					Ref.	
Monthly family income											
< 15,000 BDT	8.48 (5.44)	1.00	0.51	1.98	0.09	**0.048**	0.62	0.64	0.97	0.05	0.333
15,000–30,000 BDT	7.90 (5.14)	0.43	0.53	0.80	0.04	0.422	0.72	0.57	1.27	0.06	0.205
> 30,000 BDT	7.47 (5.21)				Ref.					Ref.	
Duration of diabetes	–	0.14	0.03	4.21	0.17	**< 0.001**	0.09	0.04	2.51	0.11	**0.012**
Taking medication for high blood pressure											
Yes	8.66 (5.24)	2.33	0.45	5.17	0.21	**< 0.001**	0.88	0.54	1.65	0.08	0.099
No	6.34 (4.99)				Ref.					Ref.	
Taking medication for high cholesterol											
Yes	8.73 (5.00)	1.65	0.43	3.88	0.16	**< 0.001**	1.21	0.50	2.43	0.11	**0.015**
No	7.08 (5.41)				Ref.					Ref.	
Cigarette smoking											
Yes	7.73 (5.00)	-0.26	0.47	-0.55	-0.02	0.583	–	–	–	–	–
No	7.99 (5.39)				Ref.					Ref.	
Physical exercise											
No	11.05 (5.44)	3.62	0.62	5.84	0.23	**< 0.001**	2.81	0.62	4.57	0.18	**< 0.001**
Yes	7.43 (5.08)				Ref.					Ref.	

Significant values are in [bold]

### Prevalence and associated factors of anxiety

Moderate to severe anxiety was reported by 22% among the participants (Fig. 1). Multivariable regression analysis showed a significant association with no formal education (β = 0.18, *p* = 0.002), prolonged duration of diabetes (β = 0.11, *p* = 0.012), taking medication for high cholesterol (β = 0.11, *p* = 0.024), and not taking part in any physical activity (β = 0.11, *p* = 0.005) (Table 3).

**Table 3:** Bivariate and multivariable regression analysis predicting anxiety.

Variables	Mean (SD)	Bivariate regression analysis					Multivariable regression analysis				
		B	SE	t	β	*p*-Value	B	SE	t	β	*p*-Value
Age		0.02	0.02	1.36	0.06	0.173	–	–	–	–	–
Gender											
Female	6.83 (4.33)	0.70	0.35	2.02	0.08	**0.044**	0.29	0.35	0.83	0.03	0.409
Male	6.13 (4.07)				Ref.					Ref.	
Education											
No formal education	8.03 (4.76)	2.31	0.46	5.03	0.24	**< 0.001**	1.76	0.58	3.05	0.18	**0.002**
Primary	6.21 (3.93)	0.49	0.40	1.23	0.06	0.218	0.46	0.46	0.98	0.05	0.326
Secondary	6.00 (0.00)	0.28	4.15	0.07	< 0.01	0.947	-0.31	4.08	-0.07	<-0.01	0.940
University	5.73 (3.9)				Ref.					Ref.	
Marital status											
Unmarried	6.26 (4.18)	-0.21	0.74	-0.28	-0.01	0.777	–	–	–	–	–
Separated	7.80 (5.69)	1.33	1.35	0.99	0.04	0.325	–	–	–	–	–
Widowed	6.71 (4.11)	0.25	0.50	0.49	0.02	0.622	–	–	–	–	–
Married	6.47 (4.22)				Ref.						
Employment status											
Unemployed	7.01 (4.47)	1.52	0.47	3.27	0.16	**0.001**	0.75	0.50	1.52	0.08	0.128
Self-employed	6.79 (4.12)	1.30	0.42	3.11	0.15	**0.002**	0.47	0.50	0.95	0.06	0.342
Employed	5.49 (3.99)				Ref.					Ref.	
Monthly family income											
< 15,000 BDT	7.21 (4.48)	1.25	0.41	3.08	0.14	**0.002**	0.52	0.52	0.99	0.06	0.321
15,000–30,000 BDT	6.52 (4.08)	0.56	0.43	1.32	0.06	0.187	0.56	0.46	1.20	0.06	0.230
> 30,000 BDT	5.96 (4.05)				Ref.					Ref.	
Duration of diabetes		0.09	0.03	3.15	0.13	**0.002**	0.08	0.03	2.66	0.11	**0.008**
Taking medication for high blood pressure											
Yes	7.00 (4.23)	1.50	0.36	4.13	0.17	**< 0.001**	0.59	0.44	1.34	0.07	0.181
No	5.50 (4.05)				Ref.					Ref.	
Taking medication for high cholesterol											
Yes	7.12 (4.09)	1.22	0.34	3.58	0.14	**< 0.001**	0.93	0.41	2.27	0.11	**0.024**
No	5.9 (4.28)				Ref.					Ref.	
Cigarette smoking											
Yes	6.18 (3.78)	-0.47	0.38	-1.25	-0.05	0.213	–	–	–	–	–
No	6.65 (4.40)				Ref.						
Physical exercise											
No	8.01 (4.61)	1.73	0.51	3.42	0.14	**0.001**	1.42	0.51	2.81	0.11	**0.005**
Yes	6.28 (4.12)				Ref.					Ref.	

Significant values are in [bold]

### Prevalence and associated factors of loneliness

Loneliness was reported by 28% of the people (Fig. 1). Being older (β = 0.13, *p* = 0.005), having no formal education (β = 0.21, *p* < 0.001), being widowed (β = 0.10, *p* = 0.020), and not taking part in any physical activity (β = 0.13, *p* = 0.005) were significantly associated with loneliness in multivariable analysis (Table 4).

**Table 4:** Bivariate and multivariable regression analysis predicting loneliness.

Variables	Mean (SD)	Bivariate regression analysis					Multivariable regression analysis				
		B	SE	t	β	*p*-Value	B	SE	t	β	*p*-Value
Age		0.03	0.01	5.67	0.23	**< 0.001**	0.02	0.01	2.80	0.13	**0.005**
Gender											
Female	4.53 (1.78)	0.22	0.14	1.58	0.06	0.116	–	–	–	–	–
Male	4.31 (1.66)				Ref.						
Education											
No formal education	5.06 (2.06)	0.92	0.19	4.93	0.23	**< 0.001**	0.83	0.21	4.01	0.21	**< 0.001**
Primary	4.29 (1.59)	0.15	0.16	0.91	0.04	0.362	0.26	0.18	1.49	0.08	0.138
Secondary	3.00 (0.00)	-1.14	1.69	-0.67	-0.03	0.502	-0.60	1.63	-0.37	-0.01	0.715
University	4.14 (1.48)				Ref.					Ref.	
Marital status											
Unmarried	4.43 (1.72)	0.16	0.30	0.54	0.02	0.587	0.16	0.29	0.55	0.02	0.583
Separated	4.70 (2.16)	0.43	0.54	0.80	0.03	0.424	0.08	0.53	0.15	0.01	0.883
Widowed	5.30 (2.02)	1.03	0.20	5.14	0.21	**< 0.001**	0.49	0.21	2.33	0.10	**0.020**
Married	4.27 (1.61)				Ref.					Ref.	
Employment status											
Employed	4.11 (1.56)	0.77	0.19	4.09	0.20	**< 0.001**	0.32	0.20	1.62	0.08	0.107
Unemployed	4.88 (1.83)	0.23	0.17	1.36	0.07	0.175	-0.05	0.19	-0.27	-0.01	0.789
Self-employed	4.34 (1.7)				Ref.					Ref.	
Monthly family income											
< 15,000 BDT	4.48 (1.88)	0.09	0.17	0.53	0.02	0.593	–	–	–	–	–
15,000–30,000 BDT	4.42 (1.66)	0.03	0.18	0.15	0.01	0.877	–	–	–	–	–
> 30,000 BDT	4.39 (1.65)				Ref.						
Duration of diabetes		0.03	0.01	2.17	0.09	**0.030**	0.01	0.01	0.42	0.02	0.676
Taking medication for high blood pressure											
Yes	4.55 (1.72)	0.36	0.15	2.41	0.10	**0.016**	0.14	0.15	0.91	0.04	0.364
No	4.18 (1.70)				Ref.					Ref.	
Taking medication for high cholesterol											
Yes	4.40 (1.63)	-0.06	0.14	-0.40	-0.02	0.688	–	–	–	–	–
No	4.46 (1.81)				Ref.					Ref.	
Cigarette smoking											
Yes	4.38 (1.54)	-0.07	0.15	-0.47	-0.02	0.639	–	–	–	–	–
No	4.45 (1.80)				Ref.					Ref.	
Physical exercise											
No	5.33 (2.02)	1.04	0.20	5.09	0.20	**< 0.001**	0.02	0.01	2.80	0.13	**0.005**
Yes	4.29 (1.63)				Ref.					Ref.	

Significant values are in [bold]

## Discussions

This study offers new data from community settings in Bangladesh, which have not been previously examined. The findings revealed that 31% of participants reported depression, 22% reported anxiety, and 28% reported loneliness. Common associated factors of all three mental states included a lack of formal education and not engaging in physical activity. Additionally, the duration of diabetes and taking medication for high cholesterol were linked to depression. Loneliness was significantly associated with older age and being widowed.

Our results are compared to other low- and middle-income, and also other areas of the world. For example, a study carried out in Ethiopia found 21.3% of patients with diabetes experience depression^39^, whilst another study^40^ found that 36.6% of diabetic patients had depression in Saudi Arabia. For anxiety, other studies including a systematic review involving 68 studies, and a multi-county study involving 15 countries, have shown respectively 28%, and 18% individuals with diabetes have anxiety^41,42^. For loneliness, a study conducted in Poland revealed that 16% of diabetic patients express intense loneliness^43^. The chronic condition of DM and its social and emotional impact can cause mental health disorders among patients with diabetes^15^.

Our study showed that lack of formal education was significantly associated with loneliness, anxiety, and depression, agreeing with previous research conducted in Poland, and Turkey^13,43^. This statistically significant correlation between anxiety and illiteracy which aligns with previous research conducted in both high income countries (USA) as well as low-income countries like Nepal^44,45^. Similarly, a study conducted in Slovenia revealed an association between low educational attainment and feelings of depression, and anxiety^46^. It is likely that lower education levels may result in limited access to information and resources and hence are more prone to mental health disorders^47^.

Being older was significantly correlated with loneliness which is in line with a previous report^48^. Our data showed a significant association between being widowed and loneliness, agreeing with findings from Poland, and the UK which indicated the lower levels of loneliness in married compared to single, divorced/ separated, or widowed patients with DM^43,49^. Studies from Brazil and Australia also showed that marital status has an association with depression and anxiety^50,51^.

The duration of diabetes was significantly associated with anxiety and depression, agreeing with previous studies conducted in China and Nigeria^52,53^, and a 15-nation study^42^. Medication for high cholesterol was linked to depression and anxiety in the present study. A prior study conducted in Malaysia revealed depressive symptoms were associated with low-density lipoprotein cholesterol, and also reported medication adherence was associated with total cholesterol^54^. Likewise, another study of Iran revealed a significant correlation of depression and anxiety with low-density lipoprotein cholesterol among patients with type 2 diabetes^54^.

Our study showed a significant association between lack of physical activity and loneliness, anxiety, and depression, highlighting the benefits of exercise for mental health, agreeing with research from Brazil, India, and Portugal, Saudi Arabia^50,55–57^. Physical activity stimulates the release of endorphins, dopamine, and serotonin, which are neurotransmitters that play a crucial role in regulating mood. The absence of physical activity was significantly associated with higher levels of anxiety in our study and others^55–57^, highlighting the significance of physical activity in fostering mental well-being. Significantly, it is important to highlight the strong association between heightened loneliness and reduced engagement in physical activity, indicating that social isolation likely has an adverse effect on participation in exercise^13,58^.

To reduce the risk of mental health problems in people with diabetes, targeted education programs for individuals, offered at community levels, especially to those with no formal education should be developed and implemented. Strategies to encourage regular physical activity and integrate comprehensive diabetes education and other healthcare approaches that address both physical and mental well-being are also essential. These interventions would be aimed at not just improving diabetic control but also the mental well-being of diabetic patients and enhancing their overall quality of life.

### Strengths and limitations

This study’s strength lies in its novel and comprehensive exploration of the prevalence and associated factors of depression, anxiety, and loneliness among individuals with type-2 diabetes in Bangladesh. It fills a critical gap in the literature, being the first of its kind in this demographic and geographic context. Its methodologies and findings offer a valuable template for replication in similar settings, enhancing mental disorder prevention and management efforts. Opportunities lie in the potential for the findings to inform targeted interventions aimed at reducing mental health disparities and improving diabetes management within communities, thereby contributing to overall health equity and well-being. There are still limitations that need to be noted. It is possible that findings may not fully capture the diverse experiences and cultural contexts of people with diabetes in Bangladesh due to the study being carried out in one geographical area. To address this, a nationwide study would need to be conducted which is beyond the reach of this study. Nevertheless, the study highlights the important risk factors that need to be addressed for a better mental well-being of people with diabetes in Bangladesh. With all cross-sectional designs, the data do not show causality. Like all large-scale surveys, self-reported measures are prone to recall bias. Lastly, the potential influence of social desirability bias on participants’ responses should be acknowledged.

## Conclusions

This is the first study to report on mental health among 600 Bangladeshi individuals with diabetes within the community. The findings indicate significant associations between loneliness, anxiety, depression, and specific factors including older age, lack of formal education, duration of diabetes, and lack of physical activity. Given the distinctive factors identified within this particular cohort, these results underscore the imperative need for tailored and strategic interventions by policy makers and healthcare providers, at grassroot levels, to target those people who are possibly underserved within the community.

## Data Availability

The datasets used and/or analyzed during the current study are available from the corresponding author upon reasonable request.
